# δ-Opioid Receptor Activation Rescues the Functional TrkB Receptor and Protects the Brain from Ischemia-Reperfusion Injury in the Rat

**DOI:** 10.1371/journal.pone.0069252

**Published:** 2013-07-02

**Authors:** Xuesong Tian, Jingchun Guo, Min Zhu, Minwei Li, Gencheng Wu, Ying Xia

**Affiliations:** 1 State Key Laboratory of Medical Neurobiology, Department of Integrative Medicine and Neurobiology, Shanghai Medical College, Fudan University, Shanghai, China; 2 Department of Neurosurgery, University of Texas Medical School at Houston, Houston, Texas, United States of America; 3 Laboratory of Molecular Neurology, Shanghai Research Center for Acupuncture and Meridians, Shanghai, China; Julius-Maximilians-Universität Würzburg, Germany

## Abstract

**Objectives:**

δ-opioid receptor (DOR) activation reduced brain ischemic infarction and attenuated neurological deficits, while DOR inhibition aggravated the ischemic damage. The underlying mechanisms are, however, not well understood yet. In this work, we asked if DOR activation protects the brain against ischemic injury through a brain-derived neurotrophic factor (BDNF) -TrkB pathway.

**Methods:**

We exposed adult male Sprague-Dawley rats to focal cerebral ischemia, which was induced by middle cerebral artery occlusion (MCAO). DOR agonist TAN-67 (60 nmol), antagonist Naltrindole (100 nmol) or artificial cerebral spinal fluid was injected into the lateral cerebroventricle 30 min before MCAO. Besides the detection of ischemic injury, the expression of BDNF, full-length and truncated TrkB, total CREB, p-CREB, p-ATF and CD11b was detected by Western blot and fluorescence immunostaining.

**Results:**

DOR activation with TAN-67 significantly reduced the ischemic volume and largely reversed the decrease in full-length TrkB protein expression in the ischemic cortex and striatum without any appreciable change in cerebral blood flow, while the DOR antagonist Naltrindole aggregated the ischemic injury. However, the level of BDNF remained unchanged in the cortex, striatum and hippocampus at 24 hours after MCAO and did not change in response to DOR activation or inhibition. MCAO decreased both total CREB and pCREB in the striatum, but not in the cortex, while DOR inhibition promoted a further decrease in total and phosphorylated CREB in the striatum and decreased pATF-1 expression in the cortex. In addition, MCAO increased C11b expression in the cortex, striatum and hippocampus, and DOR activation specifically attenuated the ischemic increase in the cortex but not in the striatum and hippocampus.

**Conclusions:**

DOR activation rescues TrkB signaling by reversing ischemia/reperfusion induced decrease in the full-length TrkB receptor and reduces brain injury in ischemia/reperfusion

## Introduction

Cerebral ischemia/hypoxia causes neuronal injury and leads to severe neurological disorders with few effective therapies available. Both clinicians and scientists have set forth enormous efforts towards exploring new clues for neuroprotection against ischemic/hypoxic injury [1,2,3,4,5]. Recent studies have demonstrated that the activation of the δ-opioid receptor (DOR) elicits a neuroprotective effect against such injuries. DOR is a type of G protein-coupled receptor and is widely distributed in the mammalian central nervous system, especially in the cortex and striatum [6,7]. Our initial work found that activation of DOR is protective against hypoxic/excitotoxic injury in the cortical neurons [8,9,10,11]. For example, DOR agonist [D-Ala2, D-Leu5]-enkephalin (DADLE) reduced glutamate-induced injury in neocortical neurons and this protection is selectively blocked by δ-, but not by µ- or κ-opioid receptor antagonists [9]. DOR activation with DADLE also increases the tolerance of cultured cortical neurons against hypoxia [10]. Furthermore, we showed that DOR provides neuroprotection against hypoxic/ischemic insults in various models including neurons under hypoxia, brain slices in hypoxia or oxygen-glucose deprivation and in vivo brain exposed to cerebral ischemia [12,13,14,15,16,17,18,19,20,21,22,23]. Intracerebroventricular treatment with the DOR agonist TAN-67 (60 nmol) significantly reduced the infarct volume and attenuated neurological deficits, while Naltrindole, a DOR antagonist, aggravated ischemic damage after forebrain ischemia in rats [12]. Similar data generated from different independent laboratories further demonstrates that DOR is indeed neuroprotective against ischemic stress in the *in vivo* models of the brain [24,25,26,27,28]. Systemic administration of DOR agonist DADLE or Deltorphin-D (variant) reduces infarct volume after transient middle cerebral artery occlusion (MCAO) [24,25]. However, the mechanisms underlying DOR neuroprotection against ischemic insults are still poorly understood.

Previous studies showed that a DOR agonist, (+) BW373U86, increased mRNA expression of brain-derived neurotrophic factor (BDNF), a member of the neurotrophin family [29,30], in the frontal cortex, and this effect was specifically blocked by Naltrindole, but not by µ- or k-opioid receptor antagonists [30]. Recent evidence also shows that BDNF plays a significant role in neuroprotection against ischemic injury [31,32]. The BDNF-mediated effect is very likely mediated through activation of TrkB, a high-affinity tyrosine kinase receptor [33,34,35]. TrkB has two major types of isoforms, i.e., a full-length TrkB protein that possesses a tyrosine kinase domain, and a truncated isoform that lacks this domain [36]. Upon activation by BDNF, full-length TrkB undergoes autophosphorylation to regulate Erk/MAPK signaling, which may increase cAMP and activate cAMP-response-element-binding protein (CREB)-regulated gene transcription, which further promotes transcription of BDNF. This is a potential positive feedback mechanism that could produce a BDNF-induced synthesis of BDNF itself [37]. On the other hand, there is also evidence demonstrating that the DOR agonist [D-Pen^2,5^] enkephalin (DPDPE) produced a dose-dependent increase in the phosphorylation of cAMP-response-element-binding protein (CREB), and this effect was reversed by DOR antagonist Naltrindole [38]. All of these results prompt us to hypothesize that the mechanism of DOR neuroprotection against ischemic injury involves a BDNF-TrkB-pCREB pathway in the ischemic brain. However, there is currently no published data in this aspect. We therefore performed this work in order to investigate such a possibility.

## Experimental Procedures

### Animals and reagents

Adult male Sprague-Dawley (SD) rats (230±5g; Shanghai Experimental Animal center and Charles River Laboratories) were used in these studies. The rats were kept on a 12 h light–dark cycle and under controlled temperature. The animal procedures were approved by The Medical Experimental Animal Administrative Committee of Fudan University and the University of Texas medical school at Houston Animal Care and Use Committee (Animal Welfare Assurance Number: HSC-AWC-11-066) and conformed to the National Institutes of Health Guide for the Care and Use of Animals in Research.

Cresyl violet, Hoechst 33258, TAN-67 and Naltrindole were purchased from Sigma (Cat: C5402, 861405, T5824 and N115-10MG). Rabbit polyclonal anti-DOR, rabbit polyclonal anti-phospho-CREB (Ser^133^), mouse monoclonal anti-phospho-CREB (Ser^133^), sheep polyclonal anti-BDNF, and donkey anti-sheep IgG-FITC were obtained from Millpore (Cat: AB1560, 06-519, 05-807, AB1513 and AP-184F). Rabbit monoclonal anti-CREB and TrkB was obtained from Cell Signaling Technology (Cat: 9197S, 4603S). Rabbit polyclonal anti-BDNF was purchased from Alomone Labs Ltd (Cat: ANT-010). Rabbit polyclonal anti-CD11b was obtained from Abcam Inc (Cat: ab75476). Anti-mouse IgG-FITC and anti-rabbit IgG-Rhodamine were purchased from Jackson Biotechnology (Cat: 115-097-003, 111-025-003). Horseradish peroxidase conjugated goat anti-rabbit/mouse IgG (H+L) were purchased from Invitrogen (Cat: G-21234, G-21040). Laemmli sample buffer, Precision Plus Protein™ Dual Color Standards and mini-protean precast gels were obtained from Bio-rad (Cat: 161-0737, 161-0374, 456-1083).

### Experimental groups

The animals were randomly allocated to 14 groups. The doses of drugs to be administered via intracerebroventricular injection were determined based on the protocol followed in previous studies [39]. The control group, comprising of rats that did not undergo MCAO, was given 10 μl of artificial cerebrospinal fluid (aCSF) containing 60 nmol TAN-67 or 100 nmol naltrindole to evaluate the effects of these drugs on cerebral blood flow and neurons (n=4 per group). In ischemic groups, 10 μl of aCSF containing 60 nmol TAN-67 or 100 nmol naltrindole was injected 30-min prior to inducing MCAO (n = 16 per group). Sham operation was performed following the same procedures as in the above groups (n = 4 per group). All drugs were injected using a microinfusion pump and infusions were delivered in ^~^5 min.

### Intracerebroventricular administration of drugs

The following drugs were used in the experiments: aCSF (pH 7.4), TAN-67 (60 nmol/10μl), and Naltrindole (100 nmol/10μl). The drugs were delivered stereotaxically into the ipsilateral lateral ventricle. In brief, guide cannulas were implanted into the right lateral ventricle (stereo coordinates: A -0.8 mm for the bregma level of anterior-posterior, L 1.4 mm for lateral, H 4.0 mm for dorsoventral on the basis of the rat brain atlas [40] and secured to the skull with dental cement. Correct placement of guide cannulas was confirmed at the time of sectioning. The lyophilized agonist and antagonist were first reconstituted (60/100 nmol/10μl) in distilled water and then in aCSF containing (in mM) 119 NaCl, 3.1 KCl, 1.2 CaCl_2_, 1 MgSO_4_, 0.5 KH_2_PO_4_, 25 NaHCO_3_, 5 D-glucose, and 2.2 urea and then filtered (0.22μm). Freshly prepared drugs were used for intracerebroventricular administration by dissolving agonists or antagonists in 10 μl aCSF and injecting into the right lateral ventricle 30 min before MCAO. The animals were sacrificed after 24 hrs of reperfusion after ischemia. The brain sections were used for immunohistochemical and western blot analysis as indicated below.

### Monitoring regional cerebral blood flow

A laser Doppler flowmeter (Periflux System 5000, PERIMED, Sweden) was used to monitor the regional cerebral blood flow. Using a stereotaxic device (SR-6N, Narishige Scientific Instrument, Tokyo, Japan) and a low speed dental drill, a burr hole of 1 mm in diameter was made over the skull at a point 1 mm posterior and 5 mm lateral to the bregma on the right side [41]. A needle shaped laser probe was placed on the dura away from visible cerebral vessels. The regional blood flow was continuously recorded beginning 10 mins before inducing ischemia until 30-min after starting reperfusion without repositioning the laser Doppler probes or the animals.

### Transient Focal Cerebral Ischemia

Rats were anesthetized with 10% chloral hydrate (360 mg/kg i.p.), and arterial blood samples obtained via femoral catheter were collected to measure pO_2_, pCO_2_ and pH with an AVL 990 Blood Gas Analyzer (AVL Co, Graz, Austria). The rectal temperature was maintained at 37 ± 0.5°C during the surgery and MCAO via a temperature-regulated heating lamp [42]. Rats with physiological variables within normal ranges were subjected to transient focal cerebral ischemia induced by right MCAO as previously described [40,42,43]. A 4-0 nylon monofilament suture with a rounded tip (diameter 0.22 mm) was introduced into the internal carotid artery through the stump of the external carotid artery and gently advanced for a distance of 22 mm from the common carotid artery bifurcation in order to block the middle cerebral artery at its origin, for 60 mins. Withdrawal of this suture restored MCA blood flow during reperfusion.

### Infarction Measurement and Fluorescence Immunolabeling

After 24 hours of reperfusion, rats were sacrificed with an overdose of 10% chloral hydrate and transcardially perfused with 0.9% saline solution followed by 4% ice-cold phosphate-buffered paraformaldehyde (PFA). The brains were then removed and post-fixed in 4% PFA for 12 h and then immersed sequentially in 20% and 30% sucrose solutions in 0.1 M phosphate buffer (pH 7.4) until they sank. Coronal sections were cut on a freezing microtome (Jung Histocut, Model 820-II, Leica, Germany) at a thickness of 30 μm at 1.60 to 0−4.80 mm from bregma and stored at −20°C in cryoprotectant solution. Sections at 1.60 to −4.80 mm from bregma were used for cresyl violet staining, and sections at 1.0 to 0.48 mm from bregma were used for immunohistochemical staining. Cresyl violet staining was performed on slices at 360-μm intervals to identify viable cells. Fluorescence immunolabeling was used to delineate the cellular localization of DOR, CREB and BDNF. Free-floating sections from each rat brain were fixed in 4% paraformaldehyde for 15 min, followed by three washes in 0.01 M phosphate-buffered saline (PBS). Sections were incubated with 0.3% H_2_O_2_ for 30 min and then placed in blocking buffer containing 10% normal goat serum and 0.3% Triton X-100 in 0.01 M phosphate-buffered saline (PBS, pH 7.2) for 30 min at 37°C and incubated with antibodies against rabbit polyclonal anti-DOR (1:200), p-CREB (1:100), CREB(1:100), and sheep polyclonal anti-BDNF(1:200), overnight at 4°C, respectively, or with antibody combinations (anti-DOR/anti-BDNF; anti-BDNF/anti-CREB; anti-CREB/anti-p-CREB). After washing with PBS, the sections were incubated with corresponding secondary antibodies (1:100) for 1 h at 37°C. Negative control sections received an identical process without the primary or secondary antibodies, and showed no specific staining. After washing with PBS, sections were then incubated in Hoechst 33258 (1μg/ml; Sigma) for 10 min in dark. Finally, these sections were mounted on glass slides and coverslipped using fluorescence mounting media. The fluorescent signals were detected by confocal laser scanning microscope (TCS SP2, Leica, Germany) at excitation 535 nm and 565 nm (Rhodamine), 490 nm and 525 nm (FITC), 352 nm and 461nm (Hoechst).

### Western Blot Analysis

A separate cohort of rats was sacrificed at 24 hrs of reperfusion after MCAO+aCSF, MCAO+TAN-67(60 nmol/10μl), naltrindole (100 nmol/10μl), and sham operation (aCSF only) (n=4 per group). The tissues of the ipsilateral striatum and cortex were quickly collected on ice, frozen immediately in dry ice, and kept at -70°C until use. Afterwards, brain tissues were homogenized in 2% CHAPS buffer because urea-saturated buffer and RIPA buffer showed similar expression of proteins whereas a higher relative expression of DOR proteins was observed after preparation using CHAPS buffer [44], [2% CHAPS (Sigma, St. Louis, MO, USA), 10 mM sodium phosphate, pH 7.2, 1% sodium deoxycholate, 0.15 M sodium chloride and protease inhibitor cocktail], and centrifuged at 12,000g for 10 min at 4°C. Protein concentration was determined by Bio-Rad protein assay (Bio-Rad, Hercules, CA). Tissue homogenates (50μg protein equivalent each) from the entire ipsilateral cortex, striatum and hippocampus of each rat were boiled at 100°C in sodium dodecyl sulfate (SDS) sample buffer for 5 mins, electrophoresed on 10% SDS-polyacrylamide gel, and transferred to the polyvinyldifluoridine membrane (Bio-Rad). Membranes were blocked with 5% nonfat dry milk in 0.1% Tween 20 (TBS-T; 2 mmol/L Tris-HCl, 50 mmol/L NaCl, pH 7.5) for 2 hours at room temperature and subsequently incubated overnight at 4°C in the blocked buffer with the 1:2000 antibody for p-CREB, CREB, and the 1:500 antibody for BDNF. After that, membranes were washed with 0.1% Tween 20, and then treated with horseradish peroxidase-conjugated anti-rabbit and anti-mouse IgG (1:5,000) for 1 hr at 37°C. Peroxidase activity was visualized with an enhanced chemiluminescence substrate system (ECL, Santa Cruz Biotechnology). Stripping filters and reprobing for β-actin was carried out for normalization. Controls for nonspecific binding were determined by omission of the primary antibody. Films were scanned with a film scanner (Image Master VDS; Amersham Biosciences Inc., Piscataway, NJ) and subsequently analyzed by measuring optical densities of immunostained bands on the film using an image-processing and analysis system (Q570IW; Leica). For each brain area, the ratios of the values obtained from ischemic and sham-operated animals were averaged from 4 different animals sacrificed at that point of time.

### Statistical analysis

Two independent and blinded investigators examined the end point assessments. Whole of the data was expressed as mean ± SE. ANOVA was utilized to analyze the difference between various groups, and p values < 0.05 were considered statistically significant.

## Results

### Effects of DOR activation and inhibition on cerebral blood flow and ischemic infarction

**Figure 1:**
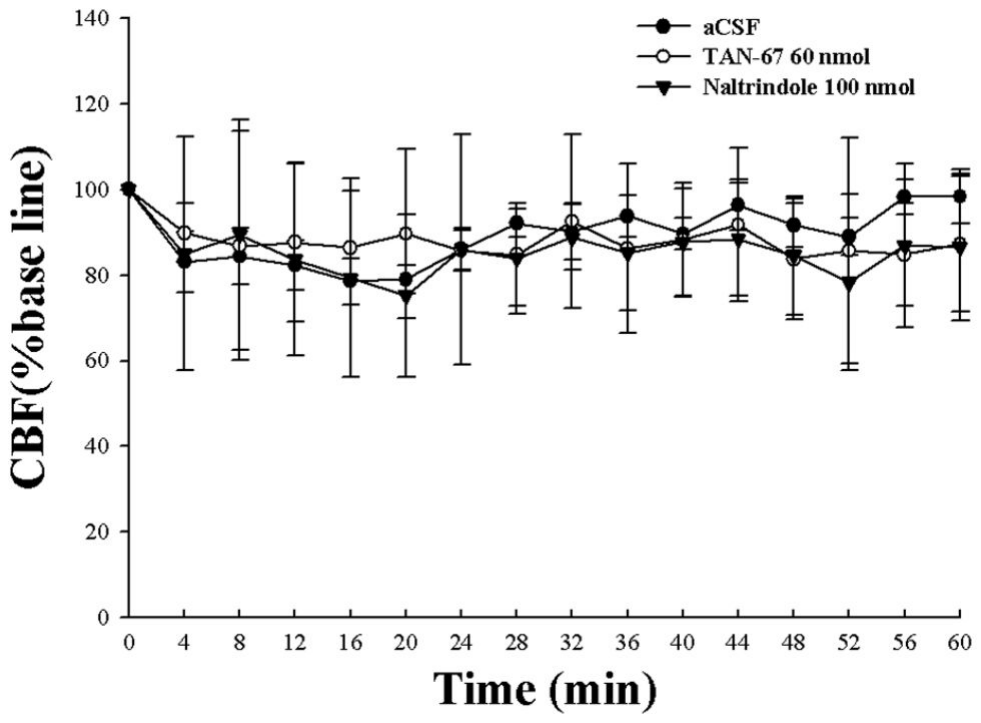
Effect of DOR activation and inhibition on cerebral blood flow. Administration of aCSF, TAN-67 or Naltrindole had no significant effect on cerebral blood flow in normal rats (*P*>0.05).

**Figure 2:**
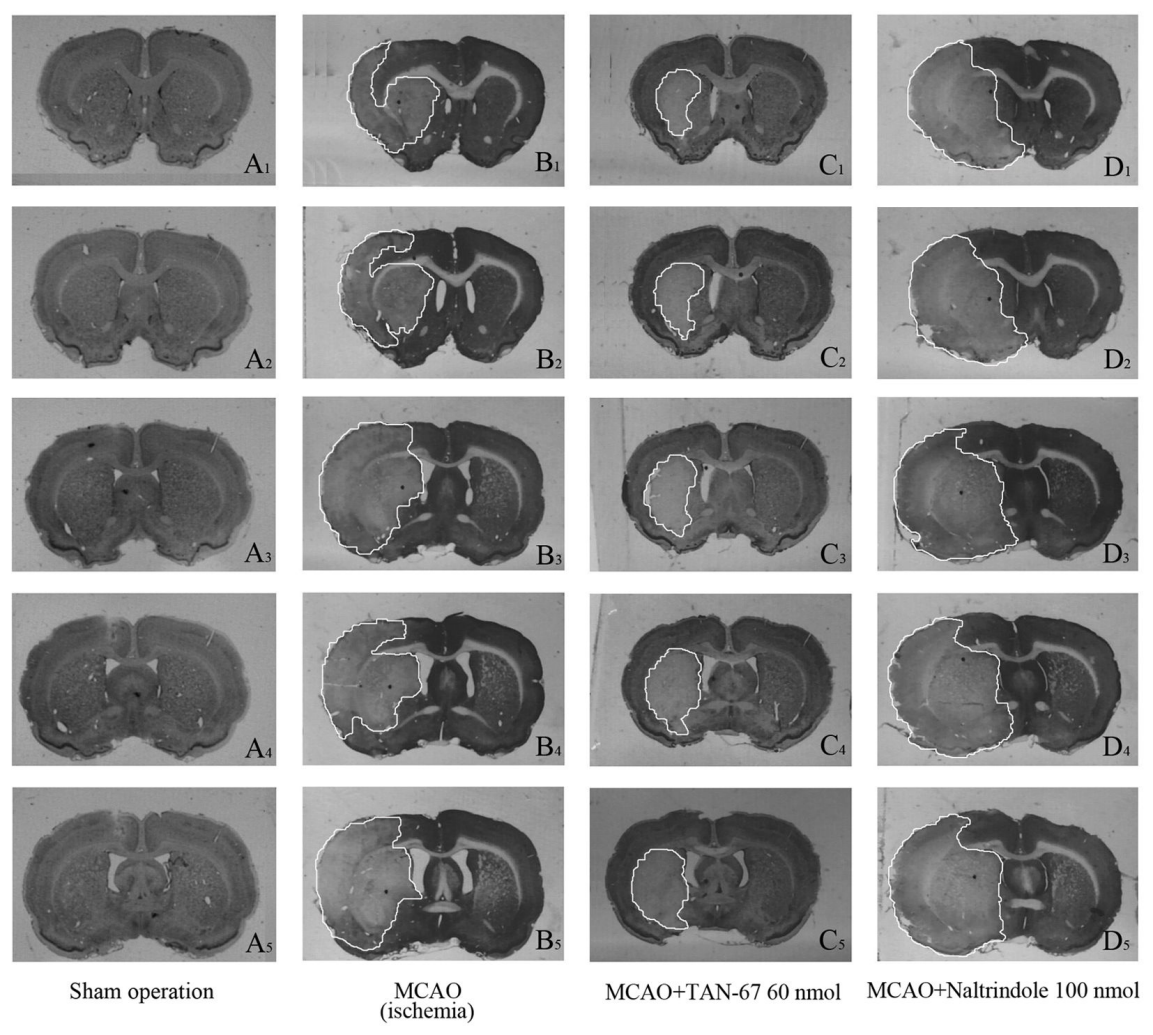
Effects of TAN-67 and Naltrindole on cerebral ischemic infarction. The area of pallor delineates the ischemic core (lateral caudate putamen), and the penumbra (frontoparietal cortex). S, Sham control. M, MCAO. Note that TAN-67 treatment decreased the ischemic size, while Naltrindole increased the infarction.

By intracerebroventricular administration, we separately applied TAN-67 and Naltrindole as well as aCSF and tested their effect on cerebral blood flow in the non-ischemic brain. There were no significant changes in the blood flow in response to DOR activation or inhibition (Figure 1). Also, these treatments had no appreciable effect on the blood flow before, during and after induction of ischemia (MCAO) (data not shown). However, DOR activation largely reduced ischemic infarction induced by MCAO, while DOR inhibition further promoted such infarction. As shown in Figure 2, all ischemic groups showed infarction typically in the striatum and cortex. Compared with MCAO only group (25.0% of the whole brain, *P*<0.05 vs. the sham control level), MCAO+TAN-67 group showed a smaller area of infarction (16.8% of the sham control level, *P*<0.05 vs. MCAO only), whereas the total infarction volume increased in MCAO+naltrindole group (35.8% of the sham control level, *P*<0.05 vs. MCAO only) (n=10).

### Effects of DOR activation and inhibition on cortical and striatal BDNF protein expression

**Figure 3:**
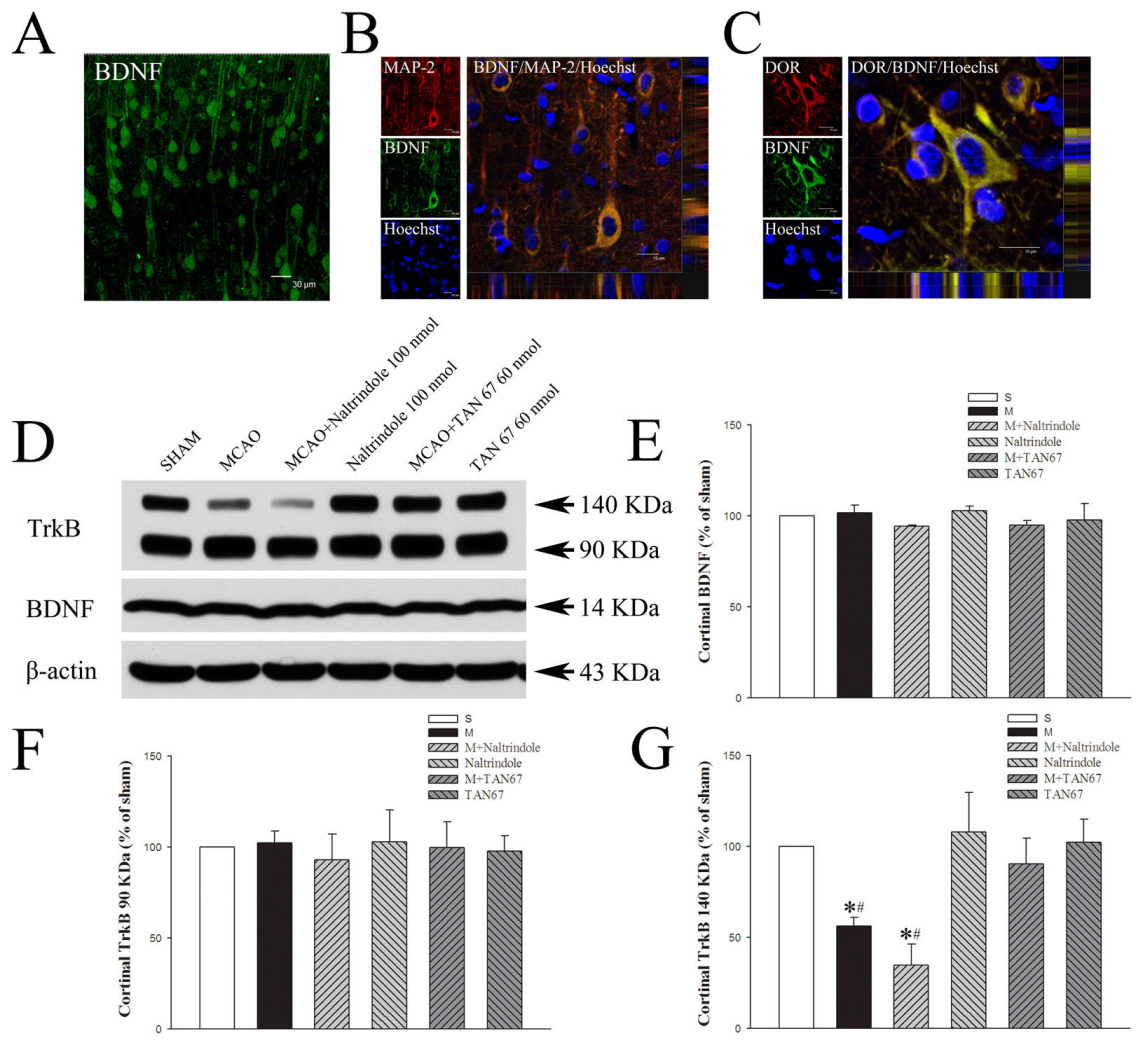
Effects of TAN-67 and Naltrindole on cortical expression of BDNF and TrkB at 24 hrs after MCAO. A, Representative fluorescent micrographs of cortical BDNF positive cells in sham group. Bar = 30 µm. B, Representative fluorescent micrographs of cortical BDNF/MAP-2 double-labeled positive cells in sham group. Bar = 15 µm. C, Representative fluorescent micrographs of cortical BDNF/DOR double-labeled positive cells in sham group. Bar = 15 µm. D, Representative Western blot images of BDNF and TrkB expression in different groups. E, Quantitative analysis of BDNF. F, Quantitative analysis of 90 KDa TrkB. G, Quantitative analysis of 140 KDa TrkB. n=4. *P<0.05 vs. the sham. #*P*<0.05 vs. M+TAN67. S, Sham control. M, MCAO. Note that MCAO significantly reduced the expression of 140 KDa TrkB but not of BDNF and 90 KDa TrkB, while DOR activation with TAN67 largely reversed the ischemic reduction of 140 KDa TrkB expression.

**Figure 4:**
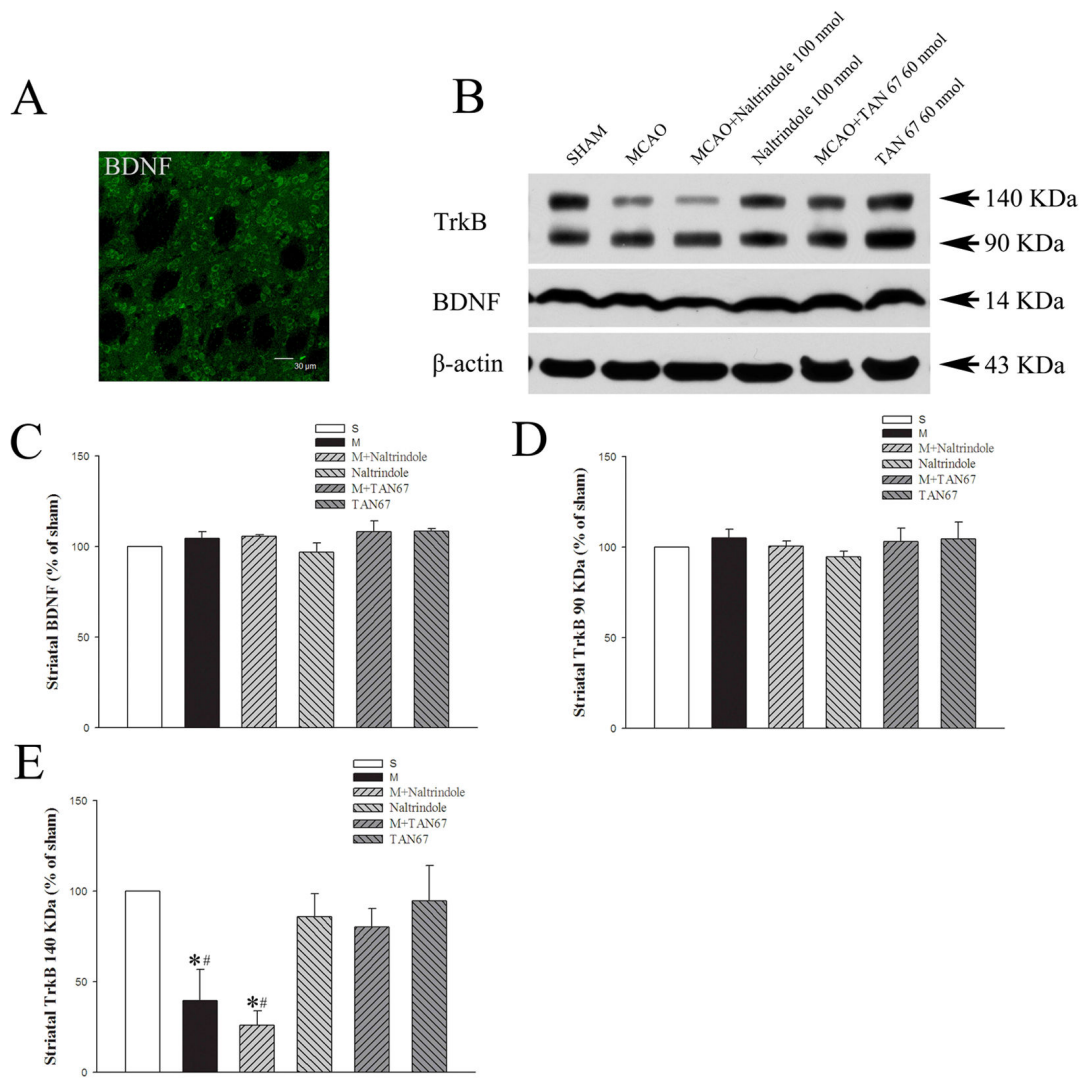
Effects of TAN-67 and Naltrindole on striatal expression of BDNF and TrkB at 24 hrs after MCAO. A, Representative fluorescent micrographs of striatal BDNF positive cells in sham group. Bar = 30 µm. B, Representative Western blot images of BDNF and TrkB expression in different groups. C, Quantitative analysis of BDNF. D, Quantitative analysis of 90 KDa TrkB. E, Quantitative analysis of 140 KDa TrkB. n=4. *P<0.05 vs. the sham. ^#^ *P*<0.05 vs. M+TAN67. S, Sham control. M, MCAO. Note that MCAO significantly reduced the expression of 140 KDa TrkB but not of BDNF and 90 KDa TrkB, while DOR activation with TAN67 largely reversed the ischemic reduction of 140 KDa TrkB expression.

Firstly, we performed fluorescence immunolabeling to determine the localizing distribution of DOR and BDNF in the cortex and striatum. The BDNF-labeled cells, exhibiting neuronal-like morphology, were found in the cortex and striatum with an abundant distribution in the frontoparietal cortex and lateral caudate putamen in sham-operated group (Figures 3A and 4A). Triple-labeled confocal images also showed that BDNF and DOR/MAP-2 protein were co-localized in the cytosol of neuronal-like cells in the frontoparietal cortex (Figure 3B and C).

After 1-hr ischemia and 24-hrs of reperfusion, BDNF expression remained at a similar level as that of the prior ischemia in the ipsilateral cortex. DOR activation or inhibition did not change the level of BDNF (101.9% in MCAO+aCSF, 95.0% in MCAO+TAN-67, 97.7% in TAN-67 alone, 94.3% in MCAO+Naltrindole, 102.9% in Naltrindole, P>0.05 among groups) (Figure 3 D, E).

Also in the striatum, we did not detect any significant change in the expression of BDNF after MCAO and after either DOR activation or inhibition in the condition of ischemia/reperfusion (104.6% in MCAO+aCSF, 108.3% in TAN-67, 105.7% in MCAO+Naltrindole, 108.3% in MCAO+TAN-67 and 97.0% in Naltrindole, *P*>0.05) (Figure 4 B, C).

### Effects of DOR activation and inhibition on cortical and striatal TrkB expression

We then examined the expression of both full-length and truncated TrkB receptors, depicted in the Figure 3. The expression of full-length (140KDa) TrkB significantly decreased after 1-hr ischemia and 24-hrs of reperfusion (56.5% of the sham level, *P*<0.05) (Figure 3 D, G). However, DOR activation greatly attenuated such a loss. As shown in Figure 3 D, G, its expression did not show any significant decrease in the MCAO+TAN-67 group after 1-hr ischemia and 24 hrs of reperfusion (90.4% of the sham level, *P*>0.05). In contrast, Naltrindole significantly worsened the MCAO-induced reduction of 140KDa TrkB protein expression (decreased to 34.8% of the sham level, *P*<0.05 vs. the sham control and MCAO+TAN-67) (Figure 3 D, G). In non-ischemic conditions, TAN-67 or Naltrindole alone did not change the expression of full-length TrkB in the sham group (102.2% in TAN-67 and 107.8% in Naltrindole, *P*>0.05) (Figure 3 D, G).

In the cortex, the expression of the truncated isoform of TrkB (90 KDa) receptors did not show any significant change (*P*>0.05) after 1-hr ischemia and 24-hr reperfusion in the ipsilateral cortex (102.4% in MCAO+aCSF, 99.8% in MCAO+TAN-67, 92.9% in MCAO+naltrindole, 97.7% in TAN-67 and 103.0% in Naltrindole) (Figure 3 D, F).

Similarly as in the cortex, the expression of 140KDa TrkB largely decreased after 1-hr ischemia and 24-hr reperfusion (39.7% of the sham level, *P*<0.05), while DOR activation with TAN-67 greatly reversed such a decrease (80.1% of the sham level, *P*<0.05 vs. MCAO alone) and DOR inhibition with naltrindole tended to worsen the MCAO-induced decrease (25.9% of the sham level). TAN-67 and naltrindole did not induce any significant change in the expression of the full-length TrkB though naltrindole tended to decrease it (94.6% for TAN-67 and 85.8% for naltrindole, *P*>0.05 vs. the sham control) (Figure 4 B, E).

The expression of truncated TrkB (90 KDa) did not show any significant difference in the ipsilateral striatum after MCAO or exposed to DOR activation or inactivation (105.0% in MCAO+aCSF, 103.0% in MCAO+TAN-67, 100.7% in MCAO+Naltrindole, 104.6% in TAN-67 and 94.6% in Naltrindole as compared to the level of the sham control, P>0.05) (Figure 4 B, D).

### Effect of DOR activation and inhibition on hippocampal BDNF and TrkB expression

**Figure 5:**
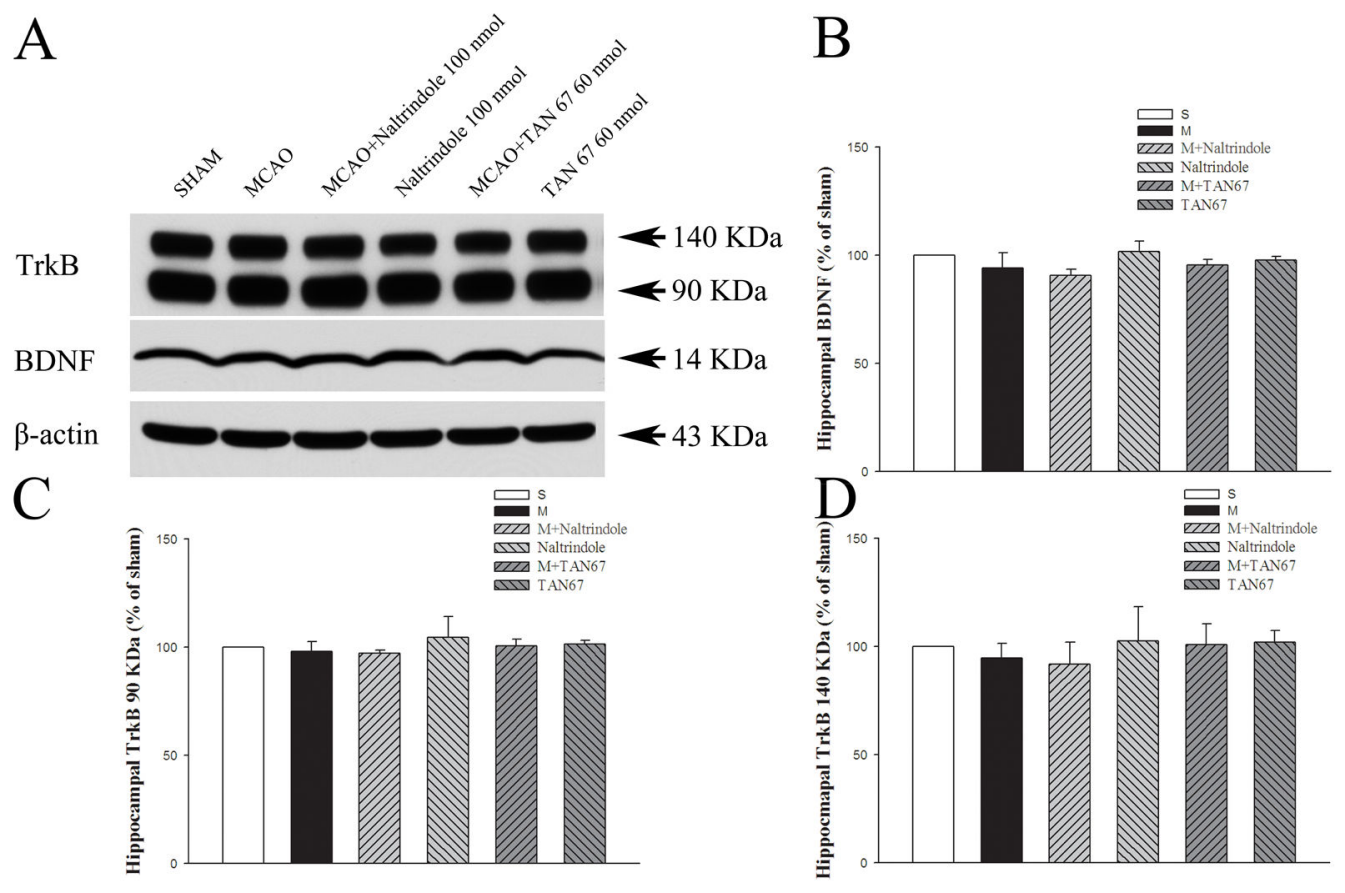
Effects of TAN-67 and Naltrindole on hippocampal expression of BDNF and TrkB at 24 hrs after MCAO. A, Representative Western blot images of BDNF and TrkB expression in different groups. B, Quantitative analysis of BDNF. C, Quantitative analysis of 90 KDa TrkB. D, Quantitative analysis of 140 KDa TrkB. S, Sham control. M, MCAO. Note that MCAO did not induce any appreciable change in BDNF and TrkB expression in the hipcampus. DOR activation or inhibition also had no significant effect on the expression of these proteins in this region exposed to MCAO.

There was no statistically significant difference (*P*>0.05) in BDNF after 1-hr ischemia and 24-hr reperfusion (94.2% in MCAO+aCSF, 95.6% in MCAO+TAN-67, 90.7% in MCAO+Naltrindole, 97.7% in TAN-67 and 101.8% in Naltrindole) (Figure 5 A, B).

In sharp contrast to the cortex and striatum, there was no statistical significance in the differences of the full-length and truncated isoforms of expression (*P*>0.05) among all groups studied (full-length TrkB: 94.6% in MCAO+aCSF, 100.9% in MCAO+TAN-67, 91.9% in MCAO+Naltrindole, 101.9% in TAN-67, and 102.6% in Naltrindole; truncated TrkB: 98.0% in MCAO+aCSF, 100.7% in MCAO+TAN-67, 97.2% in MCAO+Naltrindole 97.2% in MCAO+Naltrindole, 101.4% in TAN-67 and 104.5% in Naltrindole) (Figure 5 A, D and C).

### Effects of DOR activation and inhibition on cortical CREB/p-CREB and pATF-1

**Figure 6:**
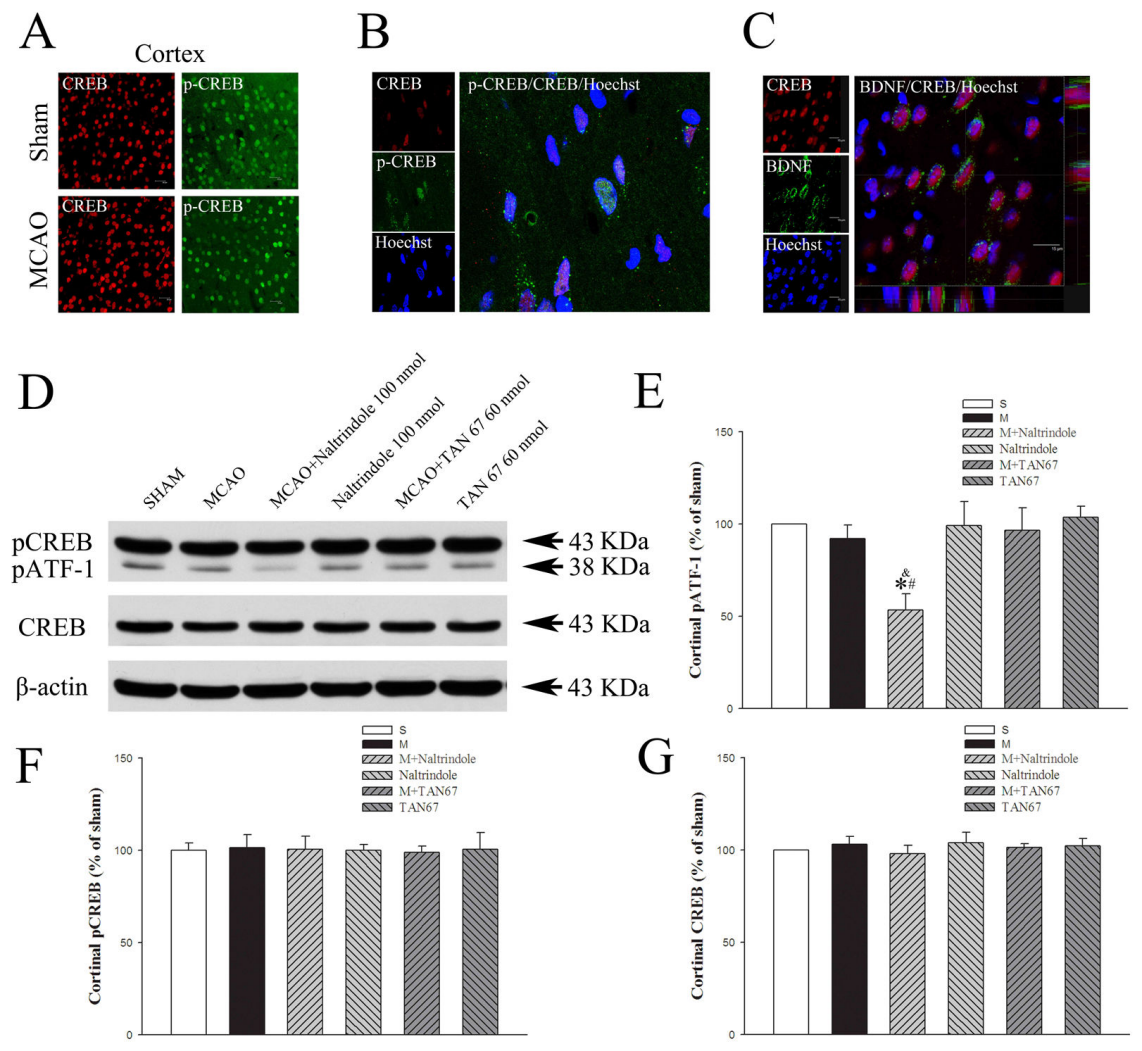
Effects of TAN-67 and Naltrindole on cortical expression of pATF-1 and p-CREB/CREB at 24 hrs after MCAO. A, Representative fluorescent micrographs of cortical p-CREB (green) and CREB (red) positive cells in sham and ischemic penumbra of the MCAO groups. Bar = 20 µm. B, Representative fluorescent micrographs of cortical p-CREB/CREB double-labeled positive cells in ischemic penumbra of the cortex after DOR activation with TAN67. C, Representative fluorescent micrographs of cortical BDNF/CREB double-labeled positive cells in penumbra. Bar = 15 µm. D, Representative Western blot images of pATF-1 and p-CREB/CREB expression levels in different groups. E, Quantitative analysis of pATF-1. F, Quantitative analysis of p-CREB. G, Quantitative analysis of total CREB. n=4. ^*^ *P*<0.05 vs. the sham. ^#^ *P*<0.05 vs. M+TAN67. ^&^ *P*<0.05 vs. the MCAO. S, Sham control. M, MCAO. Note that Naltrindole significantly reduced the expression of pATF-1 with MCAO but not of p-CREB and CREB in the cortex.

In order to explore the role of CREB/p-CREB in the DOR-mediated regulation of BDNF-TrkB signaling, we further determined their expression in the same brain regions. We observed that CREB and p-CREB were widely found in the cortex in separate staining. For example, CREB and p-CREB positive cells were abundant in the frontoparietal cortex in both sham-operated and MCAO group (Figure 6A). However, the cells co-labeling CREB and p-CREB were in a relatively small number in the cortex. Indeed, they were rarely found in the ischemic cortex in the group of MCAO alone and could be seen in the ischemic penumbra *only after DOR activation*. Figure 6B shows the co-localization of CREB and p-CREB with nuclear staining in the ischemic penumbra of the cortex treated with DOR activation with TAN-67. Triple-labeled confocal images show that CREB is co-localized with BDNF in penumbra of ischemic cortex (Figure 6C).

In western blots, we did not detect any significant change in total/phosphorylated CREB in response to ischemia with or without DOR activation or inhibition (p-CREB/CREB: 101.4%/103.1% in MCAO+aCSF, 98.9/101.3% in MCAO+TAN-67, 100.5%/98.0% in MCAO+Naltrindole, 100.6/102.2% in TAN-67 and 99.9/104.0% in Naltrindole, *P*>0.05) (Figure 6D, F and G).

As shown in Figure 6E, the pATF-1 protein level did not change in any significant way after MCAO (92.1% of the sham control level, P>0.05 vs. the sham control). DOR activation with TAN-67 did not induce any appreciable change in the he pATF-1 protein (96.5% of the sham control level, *P*>0.05 vs. the sham control). However, DOR inhibition with Naltrindole significantly decreased the pATF-1 expression in the ischemic cortex (53.3% of the sham control level, *P*<0.05 vs. that of MCAO+aCSF).

### Effects of DOR activation and inhibition on total CREB and p-CREB in the striatum

**Figure 7:**
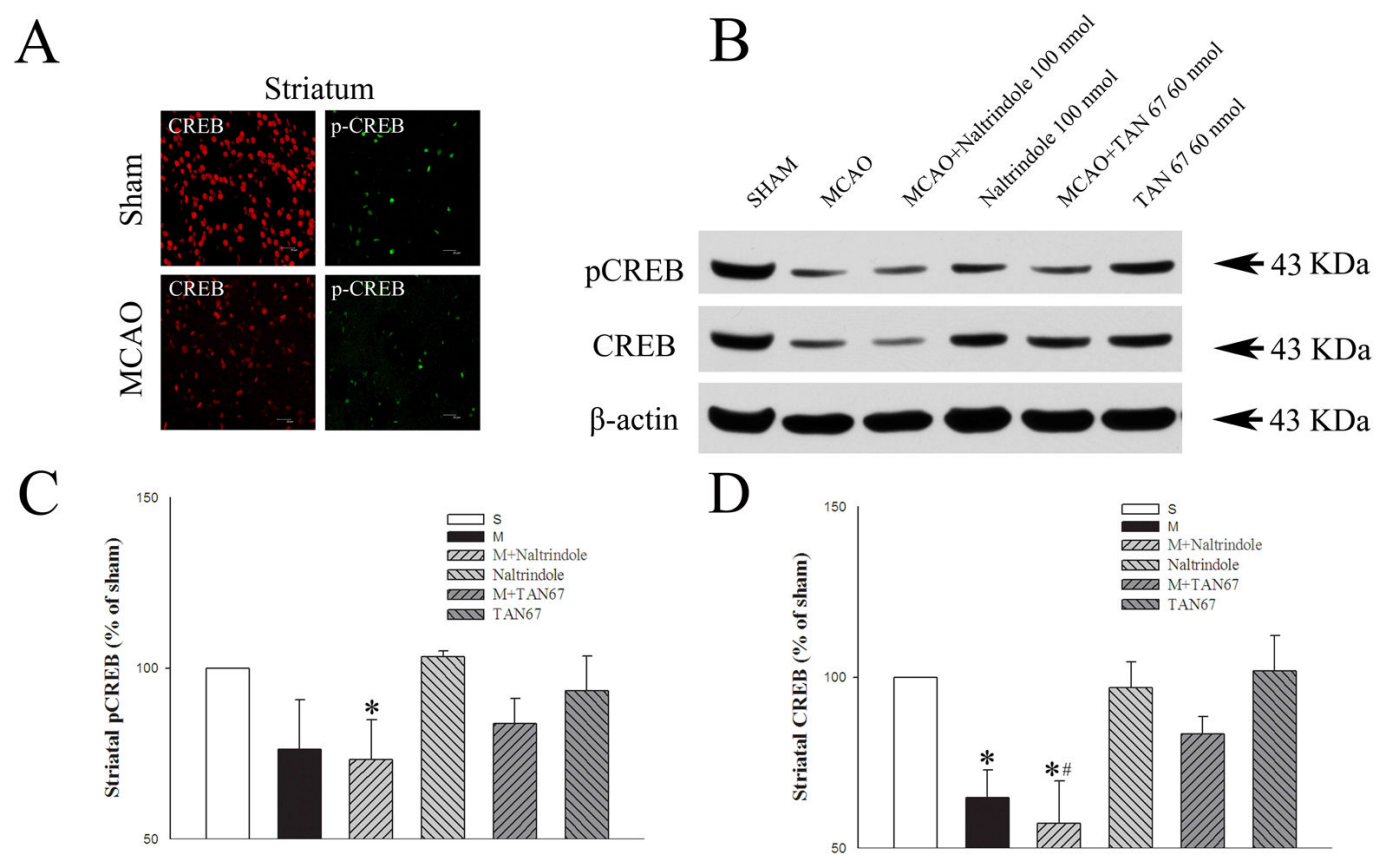
Effects of TAN-67 and Naltrindole on striatal expression of p-CREB/CREB at 24 hrs after MCAO. A, Representative fluorescent micrographs of striatal p-CREB (green) and CREB (red) positive cells in sham and the core of MCAO groups. Bar = 20 µm. B, Representative Western blot images of p-CREB/CREB expression in different groups. C, Quantitative analysis of p-CREB. D, Quantitative analysis of total CREB. N=4. *P<0.05 vs. the sham. ^#^ *P*<0.05 vs. M+TAN67. S, Sham control. M, MCAO. Note that MCAO significantly reduced the expression of CREB and p-CREB. Although DOR inhibition could not further decrease their levels, DOR activation with TAN67 tended to reverse the ischemic reduction.

As shown in Figure 7A, lateral caudate putamen were abundant in CREB and p-CREB positive cells in the sham group, but were relatively lesser populated in MCAO group. Western blotting analysis showed that p-CREB/CREB protein level decreased in the ischemic striatum of the MCAO group (64.7% of the sham control level, *P*<0.05 vs. the sham control; Figure 7). TAN-67 tended to up-regulate total CREB protein expression in ipsilateral striatum of the MCAO group though not statistically different in our sample size (83.4% of the sham control level). In contrast, Naltrindole promoted a further decrease in the expression of total CREB protein (57.1% of the sham control, *P*<0.05 vs. MCAO alone) and p-CREB (73.3% of the sham control, *P*<0.05) of the MCAO group. In contrast to the changes in the ischemic conditions, Naltrindole or TAN-67 had no appreciable effect on CREB protein level in the brain without MCAO.

We did not detect sufficient expression of pATF-1 protein in the tissue of striatum, unlike in the cortex.

### Effects of DOR activation and inhibition on CREB/p-CREB and pATF-1 expression in the hippocampus

**Figure 8:**
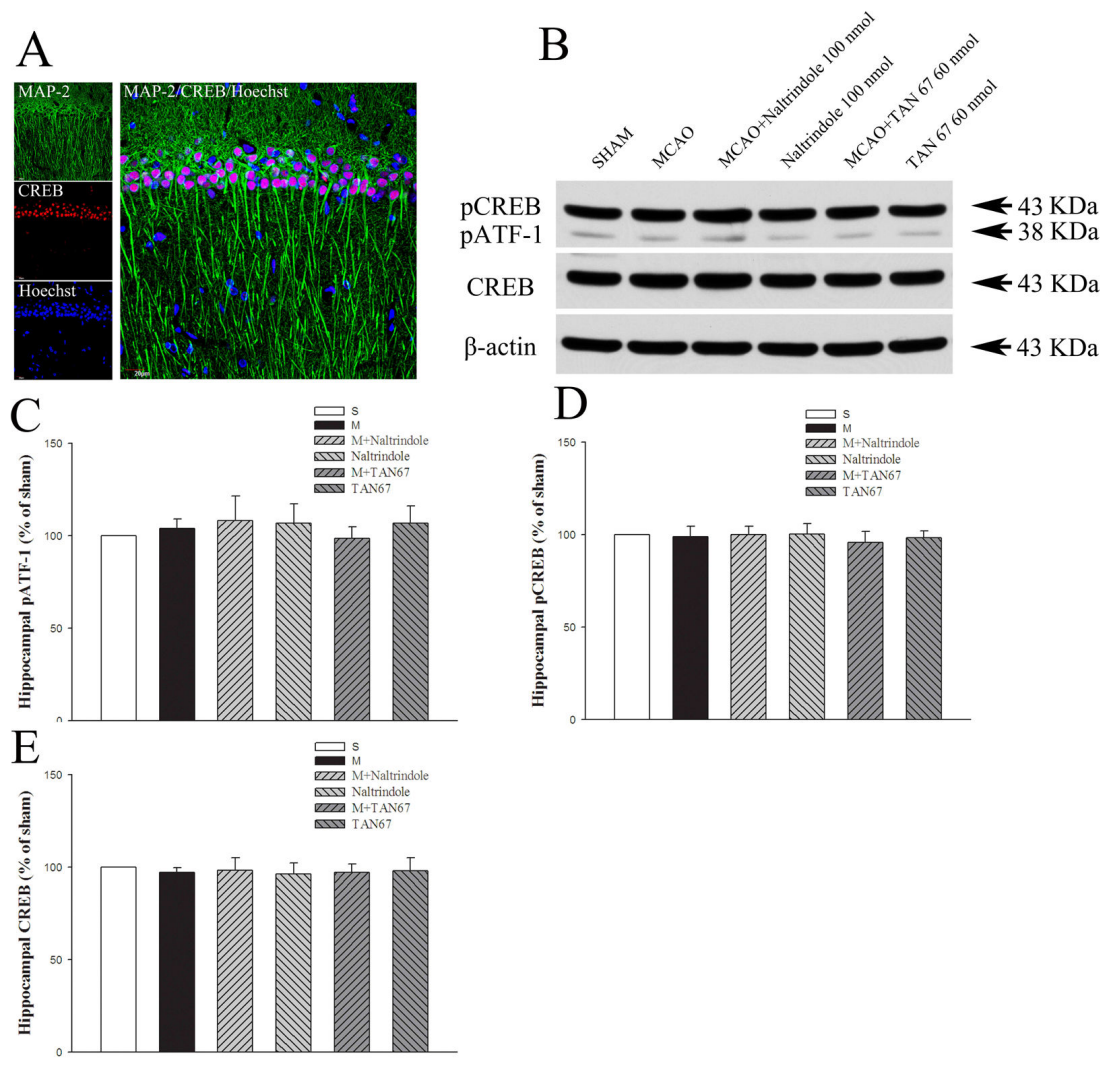
Effects of TAN-67 and Naltrindole on hippocampal expression of pATF-1 and p-CREB/CREB at 24 hrs after MCAO. A, Representative fluorescent micrographs of hippocampal MAP 2 (green) and CREB (red) positive cells in CA1 in sham group. Bar = 20 µm. B, Representative Western blot images of pATF-1 and p-CREB/CREB expression in different groups. C, Quantitative analysis of pATF-1. D, Quantitative analysis of p-CREB. E, Quantitative analysis of total CREB. N=4. Note that neither MCAO nor DOR activation had any significant effect on the expression of CREB, pCREB and pATF-1 in the hippocampus.

Using triple-labeled confocal images showing CREB and MAP 2, a neuron marker labeling mature cells exhibiting neuronal-like morphology, in the sham-operated group, we observed abundant co-localized staining in the hippocampus such as hippocampal CA1 region as shown in Figure 8A, suggesting the existence of CREB in hippocampal neurons. In western blotting studies, we found that neither total/phosphorylated CREB nor pATF-1 changed significantly in response to ischemia (Figure 8), unlike in the cortex and striatum (pCREB/CREB: 98.9/97.1% in MCAO+aCSF, 95.9/97.1% in MCAO+TAN-67, 98.3/98.0% in TAN-67, 99.9/98.3% in MCAO+Naltrindole and 100.2/96.4% in Naltrindole, *P*>0.05; pATF-1: 104.0% in MCAO+aCSF, 98.6% in MCAO+TAN-67, 106.9% in TAN-67,108.3% in MCAO+Naltrindole, and 106.7 in Naltrindole, *P*>0.05)

### Effects of DOR activation and inhibition on CD11b protein expression in the brain

**Figure 9:**
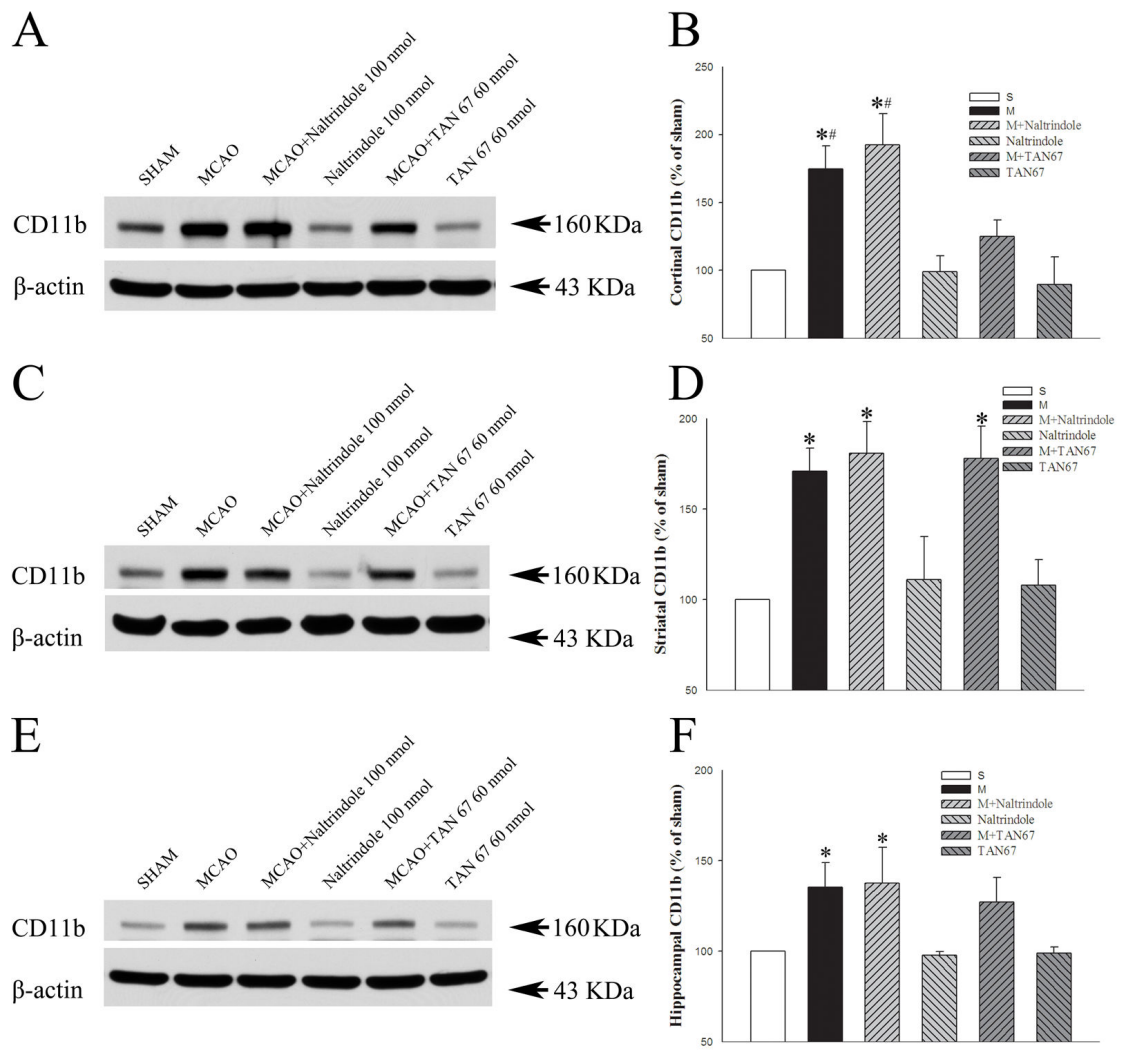
Effects of TAN-67 and Naltrindole on expression of CD11b at 24 hrs after MCAO. A, C and E, Representative Western blot images of cortical, striatal and hippocampal CD11b expression in different groups. B, Quantitative analysis of CD11b in the cortex. D, Quantitative analysis of CD11b in the striatum. F, Quantitative analysis of CD11b in the hippocampus. N=4. *P<0.05 vs. the sham. ^#^ *P*<0.05 vs. M+TAN67. S, Sham control. M, MCAO. Note that MCAO significantly increased the expression of CD11b in the cortex, striatum and hippocampus. However, DOR activation specifically attenuated such ischemic increase in the cortex, but not in the striatum and hippocampus.

We further examined whether ischemia affected CD11b expression in the cortex, striatum and hippocampus. The expression of CD11b largely increased in the MCAO group (174.6%, 171.0% and 135.2% of the sham level in cortex, striatum and hippocampus, respectively, *P*<0.05 vs. the sham control) (Figure 9). This increase was seemly enhanced by DOR inhibition with naltrindole in the cortex (192.3% of the sham level, *P*<0.05 vs. the sham control), but not in the striatum and hippocampus (180.7% and 137.7% of the sham level, respectively, *P*<0.05 vs. the sham control (Figure 9). In contrast, DOR activation with TAN-67 induced a significant decrease in the ischemia-induced expression of CD11b in the cortex (124.9% of the saline control level, *P*<0.05 vs. MCAO alone) (Figure 9), but not in the striatum and hippocampus (178.1% and 127.2% of the saline control level, respectively), suggesting that DOR activation specifically attenuated the ischemia-induced increase in CD11b expression in the cortex.

## Discussion

We have made a series of interesting observations in this work, i.e., (1) DOR activation with Tan-67 or inhibition with Naltrindole attenuated or increased ischemic infarction without any change in cortical blood flow in the ischemic hemisphere; (2) the level of BDNF remained unchanged in the cortex, striatum and hippocampus at 24 hours after MCAO, which could not be changed by DOR activation or inhibition; (3) though no significant change in truncated TrkB receptor expression, the level of full-length TrkB was greatly decreased in the cortex and striatum but not in the hippocampus by MCAO, which could be largely reversed by DOR activation, while DOR inhibition further worsened the ischemic reduction; (4) MCAO decreased total CREB expression in the striatum but not in the cortex, while DOR inhibition decreased pATF-1 expression in the cortex and reduced both total and phosphorylated CREB in the striatum; and (5) though MCAO increased C11b expression in all of the cortex, striatum and hippocampus, DOR activation specifically attenuated the ischemic increase in the cortex but not in the striatum and hippocampus. These results suggest a DOR-mediated regulation of TrkB pathway as a protective mechanism in the ischemic brain.

Although the primary cause of ischemic infarction is insufficient blood supply to the brain region involved and an increase in the blood flow can greatly relieve the ischemic infarction [45,46,47], this work demonstrated that DOR protection against brain ischemia does not rely on the regulation of cerebral blood flow because either DOR activation or inhibition had no appreciable effect on cerebral blood flow in non-ischemic and ischemic conditions. It is very likely that DOR exerts its protection through other mechanisms, especially the molecules for membrane and intercellular signaling. Although reduced or increased brain infarction may directly or indirectly affect the molecule expression levels among the groups, we found that the signaling proteins we studied changed differentially in the same group of brain tissues, e.g., no change in BDNF vs. major change in TrkB in the same MCAO group, as discussed below.

BDNF is an important neuroprotective factor that has been proven to increase the tolerance of neurons against the ischemia in both in vitro and in vivo studies. Evidence from recent studies links DOR to BDNF gene transcription and shows that the expression of BDNF is probably regulated by DOR. For instance, intracerebroventricular administration of non-peptidic DOR agonist (+) BW373U86 increased BDNF mRNA expression in the frontal cortex through DOR-mediated mechanism because the effect was blocked by Naltrindole, but not by µ- or k-opioid receptor antagonists [29,30]. Indeed, we demonstrated in this work that BDNF and DOR protein are co-localized in the cortical cytosol of neuronal cells. However, to our surprise we did not find any significant change in BDNF expression after MCAO, suggesting that BDNF is not very sensitive to ischemic stress, at least in the first 24 hours after ischemia/reperfusion. In fact, this is beneficial to the ischemic brain since BDNF is potentially neuroprotective against ischemic injury.

BDNF has been recognized to bind to TrkB receptors and thus display its signaling regulation [33,34,35]. In our measurements, there clearly existed two types of TrkB signal bands, i.e., 140 KDa and 90 KDa bands. They respectively refer to full-length and truncated TrkB receptors. BDNF activates intracellular signaling cascades through full-length trkB to induce differentiation, proliferation and survival. Activation of the full-length trkB receptors is followed by receptor dimerization and transphosphorylation on tyrosine residues [35,36,37]. The truncated TrkB receptor, though expressed abundantly in the brain, lacks the catalytic tyrosine kinase domain. It is actually a dominant-negative receptor that inhibits full-length TrkB signaling [48]. Increased truncated TrkB receptors may contribute to the reduced BDNF-TrkB signaling and may lead to neuronal injury [49]. In fact, only the full-length TrkB receptor is a functional unit and can be phosphorylated by BDNF and signal the down-stream pathways [34,36]. One of the major findings of this work is that such functional TrkB is very sensitive to ischemic stress and could decrease by 50-60% at 24 hours after ischemia/reperfusion. Therefore, it could be a crucial factor limiting the ability of the brain to overcome the ischemic stress although the level of BDNF remains at the normal level as mentioned above. Fortunately, as we demonstrated in this work, DOR activation could largely prevent such an ischemia-induced decrease, which may be an important mechanism behind the DOR-induced brain against cerebral ischemia. The role of DOR in the up-regulation of the functional TrkB receptor was further supported by the following facts: (1) DOR inhibition worsens the ischemia-induced reduction of the functional TrkB receptor and (2) DOR up-regulation of the functional TrkB receptor was evident in the DOR-rich regions of the cortex and striatum, but not in the hippocampus that has less density of DOR [7]. All of the evidence built up the ground for us to hypothesize that DOR activation protects the full-length TrkB from loss under ischmic condition. Therefore, the BDNF-TrkB pathway is likely a novel signaling mechanism for the DOR-mediated neuroprotection against ischemia stress. However, this needs to be further varified because we cannot role out the possibility that the rescured full-length TrkB receptor resulted from better neuronal survival under DOR protection.

A growing body of evidence supports an important role of the transcriptional factor CREB in mediating opioid-induced signaling [38], while phosphorylated CREB is a constitutive transcription factor and possibly mediates neuroprotection [50]. The administration of opioid drugs may regulate CREB and the subsequent signal changes in gene expression. For example, DOR agonist [D-Pen^2,5^] enkephalin (DPDPE) produced a dose-dependent increase in CREB phosphorylation and this effect could be reversed by naltrindole [38]. Tanaka et al [51,52] showed that CREB phosphorylation at Ser133 of p-CREB protein undergoes a very rapid and transient increase (3.5 hr) within the ischemic core territory following transient focal ischemia and a marked decrease in p-CREB positive nuclei at 12 and 24 hr reperfusion. In the present study, we thoroughly examined DOR’s effect on total and phosphorylated CREB proteins in all three major regions, i.e., the cortex, striatum and hippocampus, with and without ischemic condition. We used specific and well-documented CREB antibodies (Millpore, 06-519) [53,54]) that recognize both p43 phosphorylated CREB and phosphorylated ATF-1, another CRE-binding protein that has a similar sequence structure as CREB, especially in the phosphorylation domain. The total CREB was dramatically reduced in the ischemic striatum, while DOR inhibition with Naltrindole significantly reduced the expression of total CREB and phosphorylated CREB protein, implying that DOR may play a role in CREB signaling in this particular region. 。However, we did not find any relationship between DOR’s activity and total/phosphorylated CREB in the cortex and hippocampus except for the notion that DOR inhibition decreased the level of cortical pATF-1, another CRE-binding protein that has a similar sequence structure as CREB, especially in the phosphorylation domain. It seems that CREB may differentially and complexly involve in DOR signaling in different brain regions, which needs further investigation to clarify the role of CREB signaling in the DOR protection against brain ischemia. On the other hand, our data could not provide any clue as to whether CREB is upstream or downstream of BDNF, which remains unclear in the literature.

The ITGB2 subunit A, commonly referred to as CD11b or OX42, and the antibodies against this subunit are widely used as microglial markers. It recognizes both the resting and the activated microglia [55]. Microglial activation labeled by CD11b/OX42 was highly expressed in white matter regions such as the corpus callosum, external capsule, and internal capsule, rather than in the gray matter of rat brains [56]. It’s increase is an index of microglial activation [57].

Ischemia may activate microglia in the brain, which exerts both beneficial and deleterious effects on neurons. For example, activated microglia can express a variety of proinflammatory cytokines including interleukin-1β (IL-1β), interleukin-6 (IL-6) and tumor necrosis factor-α (TNF-α), which induce neuroinflammation and neurotoxicity [58,59,60]. For example, post-ischemic inflammation is characterized by a rapid activation of resident microglial cells and by infiltration of macrophages in the injured parenchyma where they release neurotoxic substances, including pro-inflammatory cytokines, chemokines, and oxygen/nitrogen free radicals to exacerbate the injury [61]. On the other side, microglia activation may result in an increase in neurotrophins such as BDNF [58], which is beneficial to the brain. Therefore, microglial activation may partially contribute to the maintenance of BDNF at a normal level in ischemic condition. This may not be the case, however, in our model because neither DOR activation nor inhibition did not change the level of BDNF despite significantly altering the level of CD11b in the cortex as well as other brain regions. We therefore rather believe that DOR activation may reduce microglial activation and decrease proinflammatory cytokines, thus attenuating ischemic injury in the cortex since DOR activation specifically attenuated the increase in CD11b in the cortex.

In summary, our data shows the importance of DOR in neuroprotection against ischemic insult. DOR activation rescues TrkB signaling by reversing ischemia-reperfusion induced reduction of the full-length TrkB receptor and down-regulates microglial activation, while having nothing to do with the regulation of cerebral blood flow. It is likely that DOR activation rescues/upregulates BDNF-TrkB-CREB signaling and suppresses microglia-released proinflammatory cytokines, thus protecting the brain against ischemic injury. Our study provides a translational clue for a potential application in clinical settings. However, the present results are limited to the pretreatment of the DOR agonist via ICV injection for transient focal cerebral ischemia. It is needed to further elucidate the effects of DOR activation in other conditions (e.g., during or after ischemia) on different models of ischemia (e.g., global ischemia). Also, it is equally important to evaluate a long-term outcome of DOR activation in ischemia/reperfusion. All these issues should be carefully addressed in future investigations.
